# DNA Damage Response in the Adaptive Arm of the Immune System: Implications for Autoimmunity

**DOI:** 10.3390/ijms22115842

**Published:** 2021-05-29

**Authors:** Theodora Manolakou, Panayotis Verginis, Dimitrios T. Boumpas

**Affiliations:** 1 Center for Clinical, Experimental Surgery and Translational Research, Biomedical Research Foundation of the Academy of Athens, 115 27 Athens, Greece; boumpasd@uoc.gr; 2 School of Medicine, National and Kapodistrian University of Athens, 115 27 Athens, Greece; 3 Institute of Molecular Biology and Biotechnology, Foundation for Research and Technology, 700 13 Heraklion, Greece; pverginis@bioacademy.gr; 4 Laboratory of Immune Regulation and Tolerance, Division of Basic Sciences, University of Crete Medical School, 700 13 Heraklion, Greece; 5 Joint Rheumatology Program, 4th Department of Internal Medicine, Attikon University Hospital, National and Kapodistrian University of Athens Medical School, 124 62 Athens, Greece

**Keywords:** DNA damage response, DNA repair, autoimmunity, adaptive immunity, lymphocytes, B cells, T cells, immune response, cytokines, therapeutic opportunities

## Abstract

In complex environments, cells have developed molecular responses to confront threats against the genome and achieve the maintenance of genomic stability assuring the transfer of undamaged DNA to their progeny. DNA damage response (DDR) mechanisms may be activated upon genotoxic or environmental agents, such as cytotoxic drugs or ultraviolet (UV) light, and during physiological processes requiring DNA transactions, to restore DNA alterations that may cause cellular malfunction and affect viability. In addition to the DDR, multicellular organisms have evolved specialized immune cells to respond and defend against infections. Both adaptive and innate immune cells are subjected to DDR processes, either as a prerequisite to the immune response, or as a result of random endogenous and exogenous insults. Aberrant DDR activities have been extensively studied in the immune cells of the innate arm, but not in adaptive immune cells. Here, we discuss how the aberrant DDR may lead to autoimmunity, with emphasis on the adaptive immune cells and the potential of therapeutic targeting.

## 1. Introduction

### 1.1. Definition and Components of DDR

**Figure 1:**
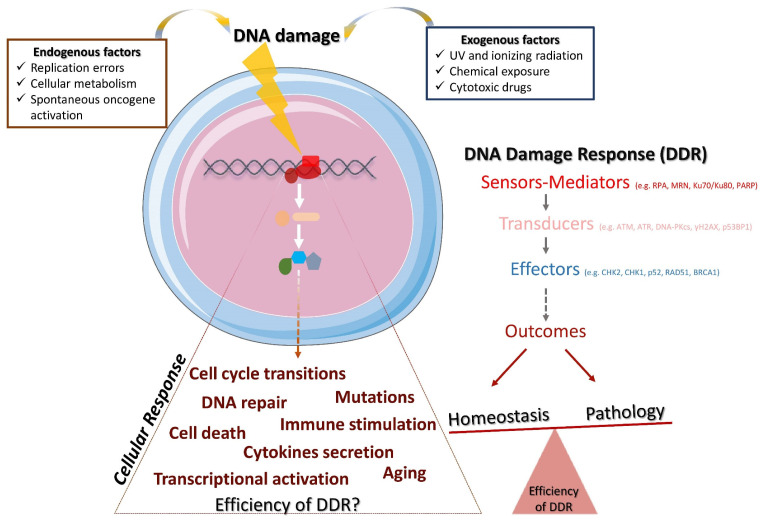
The DDR and its implications for homeostasis and pathology. Endogenous and/or exogenous factors threaten genome stability and trigger DDR. DDR is accomplished through the hierarchical activation of various molecules, namely, the DNA damage sensors and mediators, the transducers of the signaling and the effectors that carry out the outcome. Depending on the efficiency of the DDR and the cellular response, the potential outcomes may lead to the restoration of the homeostasis or to pathology.

The DNA damage response (DDR) is a mechanism that consists of multiple signal transduction pathways required to meet the challenge of passing down undamaged DNA to subsequent generations and, thus, maintaining genomic stability [1,2]. This response mechanism faces, every day, tens of thousands of damaged DNA lesions per cell [3,4,5] by activating a complex, dynamic and structured cascade (Figure 1). In this cascade of events, the DNA sensors molecules (e.g., RPA, Ku, MRN complex) recognize specific genome modifications (base mismatches, single-stranded DNA breaks, DNA adducts, double-stranded DNA breaks) and recruit the following reinforcements: (i) proteins that accumulate at the detected damaged sites and transduce the signal (e.g., ATM, ATR, DNA-PKcs, γH2AX, 53ΒBP1) and (ii) effector molecules (e.g., CHK2, CHK1, p53, RAD51, BRCA1) that carry out the critical outcome of the cascade [2,6]. These processes, consisting mainly of protein–protein interactions, are usually mediated by post-translational modifications (i.e., phosphorylation, poly(ADP-ribosyl)ation, ubiquitination, SUMOylation, acetylation, and methylation) in order to arrange spatiotemporal protein activity. For instance, ATM, a central regulator of the DNA double-strand breaks (DSBs) response, once recruited and activated, can activate p53 and checkpoint kinase CHK2 through phosphorylation in order to halt the cell cycle at the G1/S checkpoint and extend the time frame for the repair machinery.

### 1.2. DNA Repair Pathways

The DNA repair machinery consists of several pathways that usually function throughout the cell cycle, and each of them is responsible to fix a different type of DNA damage. More specifically, there are at least the following five DNA repair pathways that are frequently activated [7]: (a) homologous recombination (HR), which repairs DSBs upon the presence of a normal homologous DNA template; (b) non-homologous end-joining (NHEJ), which repairs DSBs without the need for a template; (c) nucleotide excision repair (NER), which repairs bulky DNA lesions and is important for its ability to remove the damage induced by UV; (d) mismatch repair (MMR), which repairs DNA single-strand breaks (SSBs) that occur predominantly during DNA replication and recombination; and (e) base excision repair (BER), which repairs SSBs occurred usually due to oxidation, alkylation and methylation. The potential distinct fates of the DNA damaged cells have been extensively discussed [8,9,10,11,12,13]. While the desired outcome in physiological conditions is the repair of DNA and the restoration of the cell cycle to allow the cells function properly, the outcome may also be prolonged arrest of cell cycle, cell death, tumorigenesis, secretion of inflammatory cytokines [14], and aberrant immune responses [15,16,17,18] (Figure 1).

### 1.3. Triggers of DDR

The DDR is triggered upon genome aberrations, which may occur via (a) errors during the physiological context, such as cellular metabolism, e.g., excessive formation of reactive oxygen species (ROS) and oxidative DNA damage [19]; (b) DNA replication, e.g., base mismatches [20]; and (c) inefficient activity of topoisomerases I and II [1]. The DDR operates in conjunction with the immune system to generate immune receptor diversity (such as B cell and T cell receptors) and antibodies during V(D)J recombination, class-switch recombination (CSR) and somatic hypermutation (SHM), where DSBs and/or SSBs in conjunction with the DDR/repair mechanisms are involved in the development of lymphocytes [21,22,23,24]. Notwithstanding, the DDR can also be activated by DNA-damaging agents including ionizing radiation (IR), ultraviolet light (UV), chemicals and cytotoxic drugs [25]. Overall, endogenous and exogenous insults, occurring either randomly or in a scheduled manner, jeopardize the genome integrity and activate the DDR mechanism. Depending on the mechanism’s efficiency, it is likely that the damage may result in impaired cellular function. 

### 1.4. DDR and the Immune Response

While the DDR comprises critical pathways to control cell function, the immune response comprises specialized cells responsible for mediating the organism’s homeostasis. Thus, a defective DDR in immune regulators may lead to deregulated homeostasis, and hence, pathology. A deficient or hyperactive DDR has been extensively documented during tumorigenesis and viral infections, but also in patients with autoimmune diseases and in autoimmune experimental models in vitro and in vivo, underpinning the role of the DDR in promoting autoimmunity. 

Deciphering how the DDR cross-talks with the immune system’s functions and affects its responses leading to autoimmunity, remains an open question. While much has been written on the innate components [21,26,27], the DDR involvement in the activation and function of the adaptive immune cells in the non-physiological context remains ill-defined. In general, the following two conditions make the adaptive immune cells prone to the aberrant DDR: (a) physiological processes such as V(D)J recombination, SHM and CSR, and (b) antigen-activation, where adaptive immune cells divide extraordinarily rapidly to ensure an effective immune response, thus jeopardizing the genome integrity due to DNA replication errors. Accordingly, among the blood cell populations, T and B cells have been shown to be highly sensitive to exogenously induced DNA damage [28,29].

In this review we discuss the DDR aberrancies detected in autoimmunity during random DNA damage events of the lymphocytes, and we shed light on potential therapeutic targets in this process. More specifically, we discuss the “what, why and how” of the DDR in autoimmune inflammation. We also comment on the DDR of cells that act as a bridge between the innate and adaptive immune systems, such as dendritic cells (DCs), natural killer (NK), gamma delta T (γδ T), and natural killer T (NKT) cells [3,13,30].

## 2. DDR and Immunity: A Dubious Relationship That May Culminate in Autoimmunity 

### The Early Steps and Important Findings

During the development of lymphocytes, the DDR pathways are activated in a well-planned manner, usually either by DSBs (mainly activation of NHEJ) or by abnormal base pairing (mainly activation of BER and MMR) [21]. Although the DDR and immune response mechanisms appear highly coordinated in health, herein, we discuss why and how this balance is disturbed, affecting immune cell responses and subsequently promoting autoimmunity. 

Several studies have revealed the presence of DNA damage and the aberrant DDR in either the whole-blood cells or PBMCs (human peripheral blood mononuclear cells) of patients with autoimmune diseases, but only a few have focused on the effects of the mostly implicated cell subsets, being the adaptive immune cells. In particular, autoimmunity occurs when an adaptive immune response is introduced against self-antigens. Under physiological circumstances, adaptive immunity is introduced against foreign antigens (produced by viruses or microorganisms) and is initiated by the activation of antigen-specific T cells. Eventually, it will result in the elimination of the invader through either the T cells (i.e., T cytotoxic) or the formation of antibodies by B cells (i.e., plasma cells), following an interplay with T cells, that will attack the antigens. Nevertheless, in autoimmune responses, there is an abnormal activation of the T and B cells, which leads to the release of autoantibodies against self-antigens, causing tissue damage [31]. Therefore, the adaptive immune cells have key roles in the autoimmune response. Consequently, it becomes apparent that in order to delineate the involved pathogenic mechanisms and provide insights into the disease pathogenesis, we need to profile and examine separately the involved cell populations, focusing on their unique properties.

Since the early 1980s, researchers have reported patients with autoimmune diseases presenting with aberrant DDR in their lymphocytes or PBMCs, displaying increased sensitivity to DNA-damaging agents, deficient DNA repair, and oxidative stress [32,33,34,35]. This phenomenon was considered as a breakthrough in the field of autoimmunity, but the mechanistic insight remains ill-defined to date. During the ensuing years, the need to investigate the effects of the DDR either as causal or causative of an autoimmune disease, and provide a link with immune responses, became more apparent. However, since the immune system deploys complex arrays to function, the direct interplay between the DDR and the immune response becomes extremely arduous to elucidate. Only a few studies have succeeded to provide specific mechanisms that associate autoimmune disease pathogenesis with the DDR, and most of them do not assign the observed mechanism to a specific cell population. Nevertheless, they have established a strong link between autoimmunity and the DDR, as will be outlined below.

TREX1, a significant component of the DDR, involved in the regulation of DNA repair and in the clearance of cytoplasmic DNA to prevent the activation of innate immunity, has been implicated in autoimmune responses. Respectively, cells deficient in TREX1 appear with ATM-dependent cell cycle arrest, resulting in the defective clearance of DNA in the cytoplasm [36]. TREX1 mutations, leading to the loss of its exonuclease activity, have been reported in Aicardi–Goutières syndrome (AGS) and systemic lupus erythematosus (SLE) patients [37,38,39,40]. Although both T and B cells have been shown to contribute to the autoimmune phenotype in Trex1-deficient mice [41], the reasons it promotes the inflammation only to specific organs and not to others, such as the brain and the lungs, which are often affected in SLE and/or AGS, remain unknown. Furthermore, while TREX1 has been shown to participate in systemic autoimmune diseases (i.e., AGS and SLE), its role in organ-specific autoimmunity (i.e., multiple sclerosis, psoriasis, type 1 diabetes, etc.) has yet to be determined.

Another important factor that contributes to the DDR and also influences autoimmunity is p21, which may be activated upon DNA damage via the p53 DDR effector molecule, to inhibit the cell division cycle and DNA replication, and finally promote the repair of the damaged DNA [42,43]. Interestingly, p21-deficient mice with a pre-existing mild autoreactive genetic background usually display severe lupus-like autoimmunity glomerulonephritis and promote T cell overactivation [44,45]. Notably, the in vivo overexpression of p21 directly in T cells restrained the accumulation of effector T subsets (CD4+, CD8+) [43]. Exploiting p21-deficient mice models with different autoimmune backgrounds may demonstrate contradictory results [46]. Likewise, decreased POLB activity, a crucial enzyme for the repair of damaged DNA, has been linked to SLE. In a pioneering study [47], mice expressing the hypomorphic Polb allele developed an SLE-like phenotype as a result of aberrant V(D)J recombination and a high frequency of SHM. Anti-p53 antibodies that block p53, a crucial DDR effector molecule that regulates DNA repair and cell cycle arrest, and other autoantibodies related to DNA repair components (APEX1, AURKA, POLB, AGO1, HMGB1, IFIT5, MAPKAPK3, PADI4, RGS3, SRP19, UBE2S and VRK1) have been found in the serum of SLE patients [48,49]. Of note, DSBs and deficiencies in DDR molecules, such as ATM, NBS1, MRE11A and also p53, have been observed in rheumatoid arthritis (RA) patients [50]. Screening in the serum of patients suffering from autoimmune rheumatic diseases revealed autoantibodies against the DNA repair proteins WRN and MRE11A, as well as against the critical DDR regulators Ku, DNA-dependent protein kinase catalytic subunit and poly(ADP-ribose) polymerase [51]. Collectively, these studies have provided sufficient data for the involvement of the DDR in the pathophysiology of autoimmune diseases.

## 3. Linking T Cell DDR with Autoimmunity

T cells and subsets such as helper (CD4+), cytotoxic (CD8+) and regulatory T cells (Tregs) have unique functions that shape the immune response. Dysfunctions in any T subset or the presence of autoreactive T cells have been broadly documented either as causal or causative in autoimmunity. As mentioned above, in developing T-lymphocytes DDR events operate during V(D)J recombination to generate T-cell receptor diversity (TCR) in order to recognize antigens. How the DDR normally regulates this process is outside the scope of the current review and is discussed elsewhere [21]. However, how can defects in the DDR be implicated in aberrant T cell-mediated responses in autoimmunity?

To this end, McNally et al. [52] demonstrated that antigen-activated mouse and human T cells in healthy conditions, as well as T cells in the autoimmune disease hemophagocytic lymphohistiocytosis (HLH), exhibit an increased DDR as shown by the elevated levels of classic DDR regulators γH2AX, phospho-p53 (Ser15, Ser46), phospho-ATM (Ser1981), phopsho-CHK2 (T68) and phospho-CHK1 (Ser345). The inhibition of key DDR molecules that regulate the cell cycle, such as CHK1/2 or WEE1, and MDM2, resulted in the selective apoptosis of the pathogenic activated T cells in a HLH murine model, being the CD8+ T cells, and in a multiple sclerosis (MS) murine model (such as EAE), being the CD4+ T cells. This DDR perturbation, termed as PPCA (“p53 potentiation with checkpoint abrogation”), is accomplished though the suppression of the cell cycle checkpoint and the increase in p53 activity, which does not allow the restoration of the damaged cells and leads their apoptosis. The proposed strategy by the authors does not affect other critical and immunomodulatory T cell subsets, such as naïve, Tregs and resting memory. Therefore, the authors present a potential therapeutic strategy in autoimmunity that targets only the pathogenic activated T cells. In a study published later [53], activated CD4+ T cells restored induced DNA damage compared to resting CD4+ T cells, where the unrepaired damage resulted in cell apoptosis, concluding that DDR/repair is defective in resting CD4+ T cells. The authors also provided evidence that DNA damage sensors (i.e., γH2AX, p53BP1) fail to accumulate at the damaged foci in resting CD4+ T cells, hampering the transduction of the DDR signal towards the repair mechanisms and resulting in apoptosis. Of note, the induced apoptosis relied on JNK/p73 pathway activation (and not on p53 pathway), suggesting an interplay between the DDR and other pathways to shape the cell fate. Resting T cells are non-proliferating, suggesting that the defective DDR is independent of genomic instability due to replication stress. It would be of interest to demonstrate how DDR deficiencies in resting CD4+ T cells affect cell properties with regards to cell differentiation, cytokines secretion and cell communication, and whether they contribute to autoimmune diseases, in order to provide more insights into autoimmune disease pathogenesis and treatment. Moreover, CD4+ and CD8+ T cells displayed increased DSBs in SLE patients when compared with healthy controls and RA [54], as assessed with γH2AX expression levels, which correlated with disease activity. When these cells were subjected to oxidative stress through hydrogen peroxide (H_2_O_2_) administration, the accumulated DNA damage was higher in SLE compared to the healthy controls, suggesting defects in the DNA repair mechanisms. Nevertheless, the unanswered questions that arise are as follows: Which is the underlying DDR mechanism that leads to the observed phenotype in SLE T cells? Does this mechanism drive cell behavior in SLE?

The DDR in T cells has also been associated with the cells’ DNA methylation patterns in the context of autoimmunity. For example, in SLE, T cells exhibit DNA demethylation, which correlates with T cell autoreactivity. Li et al. [55] showed that the increased expression of growth arrest and DNA damage-induced 45α (GADD45A) gene in CD4+ T cells contributes to autoimmunity in SLE by promoting DNA demethylation of CD11a and CD70, and autoreactivity. The expression levels were proportional to the disease activity. This phenomenon was exaggerated upon UV-induced DNA damage. The silencing of GADD45A resulted in increased DNA methylation of autoimmune-related genes, following a reduction in T cell autoreactivity. However, in another study [56], T cells were more prone to overactivation in mice lacking the Gadd45a gene. These mice also developed a systemic autoimmune condition resembling SLE. This discrepancy might be due to the congenital Gadd45a deficiency in the mouse model, which affects more cells than T cells, suggesting a more complex than anticipated signaling triggered by the DDR deficiency in various cells that affect the cell responses. Despite this, these articles are in agreement that GADD45A appears to be a key player in autoimmunity. Examining its expression in various cell subsets in large cohorts of autoimmune patients, and further investigating its mechanistic importance in mouse models of both induced and spontaneous autoimmunity, may shed light on the controversial evidence and establish the role of GADD45A in autoimmunity. Moreover, the mechanisms by which GADD45A may affect different organs’ homeostasis and whether it is involved in organ-specific autoimmune diseases remain to be elucidated.

Additional research has implicated defective DDR with genomic instability and T cell function in RA [50,57,58,59]. T cells in RA accumulate increased levels of DNA lesions and resist the cell cycle and repair machinery to become either hyperactive or apoptotic. Yang et al. [59] demonstrated that naïve CD4+ T cells in RA fail to activate ATM due to deficiencies in ROS production. This prevents the cells from entering the G2/M critical cell cycle checkpoint that allows the repair of damaged DNA, and thus promotes their differentiation into inflammatory effector cells (Th1 and Th17). The induction of ROS production activated efficiently the ATM pathway and decreased the cells’ immunogenicity. 

Similar to CD4+ and CD8+ T cells, the DDR mechanisms in Tregs are receiving increased attention since these cells have a pivotal role in the tumor microenvironment where DNA damage usually precedes, and in preventing autoimmunity where the DDR’s role is currently emerging. In a recent study from our team [60], the transcriptomic analysis of Tregs in MS, SLE and RA patients revealed elevated expression levels of DDR-related genes as H2AFx, TP53, CHK2 and TP53BP1. The aberrant DDR was confirmed in an experimental mouse model (EAE) of MS by the increased levels of phospho-ATM (Ser1981), p53BP1 and γH2AX proteins. This was attributed to mitochondrial oxidative stress through the production of mitochondrial (mt) ROS, resulting in cell death, accounting for the decreased numbers of Tregs found in the periphery of the autoimmune patients. Of note, Treg-specific scavenging of mtROS in vivo restrained the DDR, reduced apoptosis, and diminished the autoimmune responses. These findings assess Treg functions in autoimmunity with regards to oxidative stress and DDR, and could enable advances in immunotherapy. However, whether the aberrant DDR in Tregs detected in the aforementioned MS, RA and SLE patients is equally important and contributes to all aspects of disease pathogenesis, remains under question. Exploiting autoimmune models for these diseases that allow Treg-specific scavenging of the examined pathways and investigation of the overall disease progress, will strengthen the significance of the results. Also, considering the instrumental role of Tregs not only in autoimmunity, but also in cancer and immune homeostasis, unraveling the involved DDR mechanisms may provide novel insights into the disturbance of immune tolerance mechanisms in health and disease.

Collectively, these studies indicate that T cells exhibit an aberrant DDR in various autoimmune responses. This aberrant DDR may be directly associated with other deregulated processes that are important for cellular homeostasis, such as metabolic processes. The exact circumstances for the observed DDR manifestations and the differential roles of DDR in autoimmunity remain to be defined.

## 4. Linking B Cell DDR with Autoimmunity

The DDR events are also essential under physiological settings for the development and the cell-type specific processes of B cells. Even a slight error, during these highly coordinated and programmed processes, may lead to the aberrant DDR compromising the immune responses. Since B cells produce antibodies, and autoantibodies against the DDR/repair molecules have been reported in autoimmune conditions [48,49,51], they are positioned as the obvious suspects for the imbalanced relationship between the DDR and immune response. However, to date, the literature examining the DDR aberrancies in B cells in autoimmunity is limited.

The B cells from systemic autoimmune rheumatic disease (SARD) patients may generate autoantibodies against DDR-related proteins, suggesting that B cells respond to quiescent or lasting DNA damage preceding or during the development of overt disease. These autoantibodies are directed against Ku, MRE11A, PARP, WRN, p53, PMS1, PMS2, MLH1, and other nuclear proteins that are implicated in the DDR, and their deregulated expression levels have been associated with defective DNA repair [51,61,62]. Other autoantibodies, such as 3E10 found in SLE, affect the DDR by binding to DNA and inhibiting DNA repair. It has been proposed that the toxic effect of 3E10 on DNA is achieved when the cell is predisposed in a DNA-damaging environment [63], indicating that cells in autoimmune conditions such as lupus may be already prone to DNA damage by factors unknown so far, and the generation of DNA-damaging antibodies further exacerbates the pre-existing deregulated DDR.

Mutations that lead to the decreased expression of the DDR-associated gene POLB, identified in a GWAS, have been associated with SLE [64,65]. POLB is a DNA polymerase with a critical role in the BER pathway, therefore constituting an important mediator of the DDR outcome. Researchers generated a mouse model expressing the hypomorphic Polb allele, and highlighted that decreased Polb expression leads to SLE [41]. In particular, the mice displayed multiorgan symptoms of SLE, following the altered V(D)J recombination of B cell receptors (whereas no significant differences were detected in T cells) and increased SHM occurring in the later stages of B cell development within the germinal center (GC). Of note, both the B and T (follicular T helper) cells of GCs were increased in this mouse model, with CD4+ T cells being mostly apoptotic. This study provides robust evidence that expression derangement of DDR-associated genes involved mainly in B cell physiology can be associated with autoimmune phenotypes. Although the researchers have focused mostly on B cell properties, and less on T cells, more cells are affected by the decreased Polb expression. Therefore, it would be of interest to investigate additional cell populations affected by Polb deficiency, the effects on cell communication and their contribution to the observed autoimmune phenotype. Furthermore, since POLB appears to be an important regulator of immune responses, it would be intriguing to extend its investigation beyond the systemic autoimmune phenotype and examine the implications for distinct organs’ pathologies, therefore extrapolating its role to more autoimmune disorders.

In another recent study [66] with mechanistic insights, the differential expression of DDR-related genes in naïve B cells was sufficient to differentiate a subgroup of patients with RA with erosive disease from patients with a milder disease. In particular, this group demonstrated low expression of ATM, MRE11A and NBS1 genes of the ATM-related repair cascade of DSBs. Decreased ATM function and activation was associated with a limited BCR repertoire, an increased number of atypical B cells (CD21-/low), and the secretion of pro-osteoclastogenic RANKL and IL-6 cytokines. A loss of ATM expression has also been implicated with defects in the innate immune system enabling bacterial infections [67].

Using B lymphoblastoid cell lines from SLE patients, other studies report limited DNA repair inefficiency with regards to the DSBs repair process [68]. In particular, half of the patients (8/1) exhibited DDR defects, as shown by comet and colony survival assays after DNA damage inducing irradiation, suggesting that the repair mechanisms of damaged DNA are ineffective. Although in this study the authors demonstrate an association of defective DDR with an autoimmune disease, no specific DDR signaling pathway was described. To this end, transcriptomic analysis in several B subsets (rN: resting naïve, T3: transitional 3, aN: activated naïve, SM: isotype-switched memory and DN2: double negative) from SLE patients compared to healthy controls identified deregulated DDR pathways, such as the p53 signaling being positively enriched in all of the SLE B cells and the G2/M cell cycle checkpoint being upregulated in all of the subsets, except for DN2 B cells where it was downregulated [69]. DN2 B cells, which is a distinct population of isotype-switched cells, are enriched in SLE patients, and it has been proposed that they may contribute to SLE pathogenesis [70]. These data uncover an overall DDR deregulation across the B cell hierarchy and differentiation. Whether this deregulation affects all of the B subsets equally, and how the relationships between the differentially or similarly DDR-affected subsets may exaggerate B cell dysfunctions in SLE, remain to be addressed. Overall, these studies report the aberrant DDR outcomes to be differentially associated with many aspects of the B cell developmental processes and functions in autoimmunity, and they underscore the key involvement of the DDR in the underlying pathophysiology of autoimmune diseases. 

**Figure 2:**
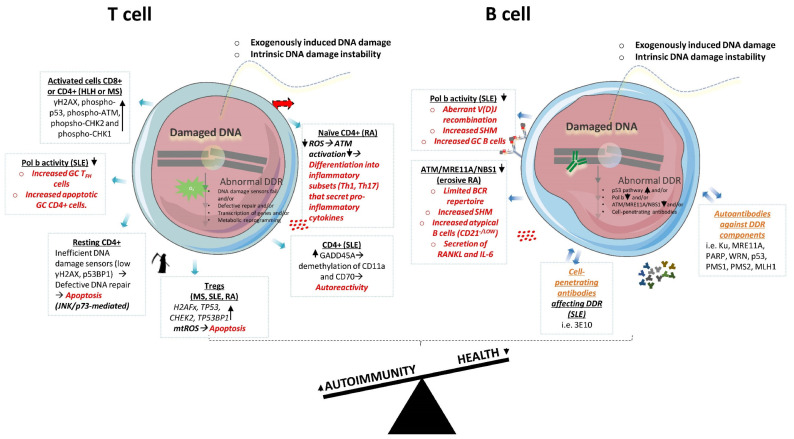
Aberrant DDR in T and B cells in autoimmune states. In autoimmunity, both T and B cells display various defects in DDR molecules, which have been associated with abnormal cellular functions. In autoimmunity, T cells display aberrant expression of DDR genes and proteins, and oxidative stress, which have been associated with alterations in cells’ differentiation into immunogenic subsets and/or increased apoptosis (marked with red color). B cells’ defects in DDR have been associated with aberrancies in V(D)J recombination, SHM, subsets formation and cytokines secretion (marked with red color). In addition, B cells in autoimmunity produce autoantibodies that may enter nucleus and affect DDR (marked with orange color). DDR: DNA damage response; HLH: hemophagocytic lymphohistiocytosis; MS: multiple sclerosis; SLE: systemic lupus erythematosus; RA: rheumatoid arthritis; TFH: T follicular helper; GC: germinal center; ROS: reactive oxygen species; mtROS: mitochondrial reactive oxygen species; Tregs: regulatory T cells; SHM: somatic hypermutation; BCR: B-cell receptor.

In summary, the aberrant DDR beyond the physiological processes of lymphocytes development have been documented in both T and B cells in autoimmunity (Figure 2). Since these cells are essential components of the immune response, these studies put the DDR forward for further investigation of the pathogenesis of autoimmune diseases.

## 5. Aberrant DDR in Innate Cells May Exacerbate Aberrant Immune Responses in Adaptive Cells: The Case of Dendritic Cells (DCs)

The generation of an immune response relies on stepwise processes among the involved cell populations. Since the innate immune response precedes the adaptive immune response, it is crucial to discuss, for the needs of this review, the predominant innate cell component that primes adaptive immune cell functions, being the antigen-presenting (APC) dendritic cells (DCs). The DCs interact and stimulate antigen-specific T cells, which, accordingly, activate B cells. Consequently, a putative dysfunctional DC, due to aberrancies at its DDR mechanisms, could have an escalating impact throughout the adaptive immune response.

In this direction, researchers have identified the DCs from Nlrp3-/- and Caspase 1-/- mice to exhibit reduced levels of DNA damage and p53-induced apoptosis as a result of effective DNA repair mechanisms, following exposure to DNA-damaging agents such as oxidative H_2_O_2_ and genotoxic MSU crystals [71]. Of note, high expression levels of Nlrp3 and Caspase 1 have been associated with a plethora of autoimmune diseases [72,73]. Therefore, these data imply that the DCs may require decreased Nlrp3 and Caspase 1 expression levels to have an efficient DDR, yet many autoimmune patients are characterized by increased levels and, accordingly, they may undergo a defective DDR. In brief, the inflammation caused by NLRP3/Caspase 1 activation may fuel the DDR in DCs, which in turn exhibit increased immunogenicity. Moreover, the DCs, following the inhibition of the key DDR orchestrator ATM, either genetically or pharmaceutically display delayed maturation, reduced T-cell activation and increased apoptosis, suggesting that ATM is critically involved in their development and functions [74]. A loss of ATM function has been implicated in severe autoimmune conditions [66]. In this study [74], ATM function is considered as DNA damage-independent, since DNA damage levels were not reported, suggesting that the DCs might have not be subjected to actual DNA damage. Collectively, these studies document the regulatory function of the DDR or DDR-related molecules on the DCs development; however, they do not provide direct indications for the effects on the subsequent cell interactions within the adaptive immune branch.

The DC-T cells interactions are followed by the synergy between T and B cells, which is essential for the development of appropriate antibodies and the efficiency of adaptive immune response. Defects in their interaction may lead to autoimmune responses [75]. Should one of these contributors transmit defective signals to the other due to the aberrant DDR issues, the DDR’s defective outcome is propagated through various pathways and cells, and may result in dire consequences in later states, in a cascade-like fashion. For example, in RA patients there is a PD-1hiCXCR5-Bcl6low T peripheral helper (TPH) cell population that infiltrates inflamed synovia and is involved in the priming of B cell responses [76]. Notably, p53 is able to suppress the CHCR5 chemokine receptor through the inhibition of NF-kB activity and BCL6, reinforcing the DDR [77,78]. Thus, the DDR in T cells may both affect their responses and determine the B cell response. This may explain why depletion therapies of a single-cell population (i.e., B cell depletion therapies) may not be sufficient for a robust therapeutic response. To that end, further research is required to decipher the DDR-dependent mechanisms of crosstalk among DCs, T and B cells.

## 6. The Role of DDR in Other Cells of the Adaptive Immunity: NK Cells, γδ Τ and NKT Cells

The NK, γδ T and NKT cells represent a bridge between the innate and adaptive responses [79,80] since they are of lymphocytic lineage with innate features. Their involvement in autoimmune diseases has been described both as disease-controlling and disease-promoting [81,82,83]. NK cells have germline-encoded antigen receptors, and therefore they do not undergo V(D)J recombination, whereas γδ T and NKT cells express TCRs derived from V(D)J recombination. All three of the subsets influence T and B cells and their effector actions.

A normal DDR is important for the development of NK cells, and RAG enzymes’ functions are involved in DDR outcomes [84]. Of note, RAG enzymes (RAG1 and RAG2) have been initially studied for their critical involvement in V(D)J recombination by introducing DNA DSBs [85,86]. Notwithstanding, the researchers studied NK cells that do not undergo V(D)J recombination and revealed an additional DDR role for the RAG enzymes with regards to NK cells’ expansion, survival and responses. More specifically, RAG deficiency in murine NK cells displayed increased γH2AX levels at steady-state, and was associated with an impaired DDR characterized by DNA-PKcs, Ku80, Chk2 and ATM reduced gene expression. Importantly, RAG deficiencies (RAG1/RAG2) have been associated with autoimmunity [87,88,89], suggesting that the aforementioned DDR outcomes may be implicated in the pathogenesis of autoimmune diseases. 

Another DDR-related molecule that is crucial for NK cells’ functions, and also for γδ T, NKT and several T cell subsets, is the NKG2D receptor whose ligands are modulated by ATM/ATR DDR signaling [90,91]. The NKG2D receptor and ligands have been implicated in numerous autoimmune diseases [92,93,94,95,96,97,98], since their aberrant expression may lead to the activation of autoreactive effector cells and trigger autoimmune responses. A role of the DDR in the NKG2D receptor and ligands’ deregulated functions has not been clearly reported in autoimmune diseases. Yet, human and mouse data in the context of cancer [91] suggest that NKG2D ligand overexpression detected in tumor cells may be due to chronic activation of the DDR in order to trigger the immune system. Finally, Swann et al. [99] reported that NKT cells exhibited a tumor-suppressive role in cancer caused by the p53 deficiency in mice, implying a link of NKT modulatory functions in concert with DDR mechanisms.

Taken together, these studies support the contribution of the DDR to NK, γδ T and NKT cells’ immune responses, adding a layer of complexity that involves various immune cell populations being affected by the DDR components. However, a direct causative link of this contribution to the pathophysiology of autoimmune diseases remains to be established.

## 7. DDR and Cytokines in Autoimmunity

### 7.1. DDR May Lead to Exaggerated Cytokine Production and Promote Autoimmune Inflammation 

Cells produce cytokines that orchestrate all facets of an immune response. In particular, cytokine production allows the communication among cells, and regulates the development and activities of particular cell populations. Pro-inflammatory cytokines contribute to the pathogenesis of autoimmune diseases. Interestingly, the production of pro-inflammatory cytokines, such as IL-6, TNF-α, IFN-γ, has been associated with genome instability following the DDR [14,100]. These cytokines are secreted by a wide range of cell types, including T and B lymphocytes. 

In the case of IL-6, Rodier and colleagues [14] demonstrated in vitro that the IL-6 response required the persistent activation of the DDR signaling though the DDR proteins ATM, CHK2 and NBS1 (NBS1 expression is usually required for optimal ATM activity). Although, in this study, researchers used mainly fibroblasts, IL-6 is also produced by adaptive immune cells (i.e., B cells) and is essential for the maturation of B cells into antibody-producing cells. In other words, the DDR occurring either in B cells per se or in other cells that interact with B cells (such as T cells), may increase IL-6 secretion, which stimulates autoantibody production leading to autoimmune disease.

### 7.2. Cell Free DNA may Induce Cytokine Production

Cell-free DNA from apoptotic cancer cells integrates into the genome of neighboring healthy cells to induce cytokine production (such as IL-6, TNF-α and IFN-γ), suggesting that the aberrant DDR and inflammatory response are closely linked pathologies [100,101]. In a physiological context, extracellular DNA does not drive genome instability as long as (a) its degradation is fast and (b) it does not integrate into the genome. In general, foreign DNA can integrate into the genome by either exploiting sequence homology-dependent means or by performing illegitimate integration via homology-independent processes [102]. However, the involved mechanisms that drive the integration into a specific cell are unclear. Nevertheless, either the integration being homology-dependent or -independent, the involved machineries require adjustments to the cell cycle employing the DDR system. Interestingly, high levels of cell-free DNA have been reported in autoimmune diseases, such as SLE, probably due to defective clearance [103,104]. Since, among the immune cell populations, T and B cells display extremely rapid division rates deviating from regular cell cycle checkpoints, they become susceptible to genome instability, facilitating DNA integration into these cells. Therefore, the release of cytokines observed in autoimmune conditions could be associated with the genome instability of T and B cells, caused from the integration of the cell-free DNA into their genome. Thus, the aforementioned aberrancy may follow the defective extracellular DNA clearance seen in autoimmune diseases.

Overall, these studies indicate that the DDR contributes to excessive cytokine release, promoting immune responses towards autoimmunity. Yet, the involvement of particular adaptive immune cells and the DDR signal have not been characterized thus far. Nevertheless, future studies in this field may offer knowledge with tremendous impact on autoimmunity, considering the plethora of cytokines already targeted in the clinic to treat patients with autoimmune diseases [105,106].

## 8. Therapeutic Manipulation of DDR in Autoimmunity

### 8.1. Lessons Learnt from Cancer

Genomic instability and DDR deregulation have been extensively investigated in cancer, where they have been linked to tumorigenesis. The development of DDR-targeted drugs in cancer, either genotoxic or against proteins, aims to attenuate the DNA repair ability of the tumor cells in order to facilitate radiotherapy to eliminate the tumor cells, or to promote synthetic sensitivity or lethality (SSL). In particular, synthetic sensitivity diminishes cell divisions, which, when accompanied with additional therapeutic agents such as cytotoxic drugs, may result in cell death. Olaparib [107] is an FDA-approved PARP inhibitor developed in BRCA1/2-defective cells. PARPs are enzymes that sense the DNA damage and contribute to the activation of repair pathways. Their inhibition results in the accumulation of DSBs, which are supposed to be repaired by the HR repair pathway. In cells with BRCA1/2 gene defects, as in the case of ovarian cancers, HR signaling is deficient, and therefore the cells end in cycle arrest and reduced cell viability via synthetic lethality [108].

Pearl at al. [109] have performed an extensive systematic computational analysis to identify direct druggable opportunities in the DDR protein components exploiting large-scale genomic and expression data for 15 cancers, and they discovered possible targets for all of the major DDR pathways. It is important to note that the DDR-based therapeutic strategies do not directly affect the DNA structure and, therefore, they are considered to be nongenotoxic to the patients. Of note, among the characterized current drugs or novel targets, there are the DDR proteins whose deregulation is also implicated with autoimmune diseases, such as CHK1, p53, PARP, MRE11A, ATM. 

### 8.2. DDR Targeting in Autoimmune Diseases

In this context, the question that arises is how these targets could be modulated therapeutically in autoimmune diseases. One approach may be to inhibit their expression in pathogenic cells (e.g., antibody-producing B cells in SLE) and test whether this restrains their pathogenic phenotype. Nonetheless, many of these DDR targets have been found downregulated (instead of upregulated as in the case of many cancers) in autoimmune conditions, suggesting that the suppression instead of the upregulation of their expression may be harmful. Yet, this downregulation is usually observed either in the mRNA or the total protein’s levels and does not always mirror the active form (e.g., phosphorylated proteins) of the protein, since most studies have documented numerous phosphorylated DDR-involved proteins overexpressed in autoimmunity. In particular, DDR signaling and outcome is transduced and determined by a series of successive post-translational modifications of the involved molecules. It is likely that there is a negative regulatory loop for the expression of such genes. Targeting the active instead of the inactive DDR proteins could be a reasonable approach. 

A DDR-based therapy to suppress the immune response in antigen-activated T cells in the human autoimmune diseases HLH and MS has been proposed by McNally [52]. After the screening of DDR-altering small-molecule compounds, they provided evidence in vivo and in vitro that a combination of therapeutic strategies enhancing the p53 DDR pathway (via targeting MDM2) and attenuating the cell cycle checkpoints (via targeting CHK1 or WEE1) results in the selective elimination of pathological T cells and the treatment of the autoimmune murine models.

In a more forward approach, radiation therapy, which is broadly used for cancer treatment to eliminate cancer cells and enhance the efficiency of other immunotherapies, could be exploited, at lower levels than the one used in cancer, in autoimmunity for the manipulation of the cells’ immunogenicity. More specifically, it has been recently shown that radiation therapy may induce TREX1 activity to degrade the accumulated DNA in the pathogenic cells and reduce their immunogenicity [110]. Since (a) B and T cells are more sensitive to irradiation [28,29,111]; (b) TREX1 activity is decreased in various autoimmune conditions [37,38,39,40]; and (c) B and T cells’ immunogenicity is a key contributor to the autoimmunity, researchers could tackle the question whether low-dose irradiation could be exploited to ameliorate autoimmunity.

In light of these developments, we speculate that in the near future selective aspects of autoimmunity may be exploited for anti-tumor therapies. In this direction, the existence of autoantibodies that penetrate into the cell nuclei and threaten the genome integrity and cell viability, has been well documented during the last years [62]. For example, autoantibodies, such as 3E10, may have the ability to selectively damage and affect the DDR of the cells that are susceptible to genome aberrations. How these autoantibodies contribute to SLE pathogenesis is not clearly understood. It is possible that they exaggerate the inflammation and the production of other autoantibodies following the signaling perturbation they provoke into the cell by binding to DNA [62]. It is therefore expected that SLE patients have an increased risk of certain types of cancer [112,113,114,115]. Accordingly, this type of cell-penetrating autoantibody that damages DNA may have a therapeutic potential against cancer [116]. Specifically, since these autoantibodies do not appear to affect healthy cells, but only those that are predisposed to DDR defects, they could be exploited for (a) the selective elimination of tumor cells and (b) the treatment of immunogenic cells in the context of autoimmunity that demonstrate DDR defects, as in the case of adaptive cell subsets reviewed here, by transporting molecules able to restore the cellular properties [117].

In summary, targeting the DDR aberrations in autoimmunity could be accomplished within the following frames: (a) targeting the DDR molecules with abnormal expression in specific cell populations, where DDR plays a role beyond the physiological cellular functions resulting in the formation of immunogenic cells; and (b) since the DDR per se is critical for the cell survival and expansion, targeting the DDR in immunogenic cell populations—even if the DDR has not been associated with the cell’s unfavorable functions—in order to eliminate the pathogenic cells. Since the adaptive immunity is a fundamental driver of autoimmunity, targeting selective adaptive immune cell types could result in improved therapeutic outcomes in the treatment of autoimmune diseases. The use of cell-penetrating antibodies that may transport therapeutic agents into the damaged cells, may also hold a therapeutic potential in autoimmunity. In this direction, there is an emerging need for developing suitable animal models that reliably reflect the pathogenesis of autoimmune diseases and allow the pre-clinical experimentation of cell-based therapeutic strategies.

## 9. Synthesis and Concluding Remarks

In this review, we discussed the aberrant DDR in autoimmunity, focusing on the cells of the adaptive immune branch and on the cells that bridge the innate with adaptive immune response (see key findings summarized in Table 1). Further research is needed to expand our knowledge regarding the role of the DDR in the pathogenesis of autoimmune diseases. To this end, the generation of suitable in vivo models of autoimmune diseases, where the cell-targeted therapeutic approaches of the DDR could be examined, is of outmost importance (see research agenda, Table 2). Linking the DDR molecules’ dysfunction with cell behavior during the autoimmune response is also essential. Moreover, the investigation of the effects of the aberrant DDR on the interactions and signals among the immune cells may explain the overall derangements observed during the immune response in autoimmunity. On the other hand, autoimmune features, such as cell-penetrating autoantibodies that violate cells with pre-existing genome instability, may be used for engineering antibodies for targeted molecular therapy. Finally, specific targeting of the DDR-involved molecules could be a new frontier in the management of autoimmune diseases.

### Open Questions

Considering the emerging role of DDR in adaptive immune cells and its implications for autoimmunity, some challenges are listed below:

1. Comprehensive understanding of the DDR cascade in B and T cells in autoimmune diseases:
  (a) Do DDR alterations in lymphocytes drive their autoreactive phenotype (e.g., plasmablast formation, antibody production, inflammatory cytokine secretion)?
  (b) How do these alterations imprint on the inflammatory cascade driven by autoreactive B and T cells?
  (c) Does reversing the DDR aberrancies in lymphocytes restore homeostasis, leading to the remission of autoimmunity?
2. Delineation of the DDR molecular pathways in lymphocytes in autoimmunity:
  (a) Combine genomic, proteomic and epigenetic technologies to uncover new checkpoint molecules on the DDR aberrancies in autoreactive lymphocytes, which will provide further insights for future therapeutic interventions.
  (b) Considering that metabolism orchestrates B and T cells functions, revealing a potential crosstalk of metabolic and DDR pathways operating in autoreactive lymphocytes may provide further knowledge for the disturbed self-tolerance.
  (c) Can early DDR events drive therapeutic decisions in autoimmunity?
3. Do the DDR pathways function differently in multi-organ damages in systemic autoimmunity (e.g., SLE, AGS, RA) compared to organ-specific autoimmunity (e.g., MS, psoriasis, type 1 diabetes)? What are the distinct pathways involved in each disease?
4. Do current therapeutic regimens affect the DDR in adaptive immune cells? Do they deregulate the DDR? For instance, JAK inhibitors that are used for cytokine targeting may attenuate the DDR and increase DNA damage cargo [118], and thus influence the resistance to therapy.

## Figures and Tables

**Table 1:** Key findings in studies addressing DDR of adaptive and adaptive-related immune cells.

	Cell Type	Autoimmune Context	DDR Mechanism	Proposed Therapeutic Targeting	Reference
**Main adaptive immune cells**	CD8+ T	HLH	Increased γH2AX/phospho-p53, phospho-ATM, and phospho-CHK1/2 (activated healthy T cells)	PPCA therapy eliminated only pathological CD8+ T cells	[52]
	CD8+ T	SLE	Increased γH2AX/defective repair after induced DNA damage		[54]
	CD4+ T	MS	Increased γH2AX/phospho-p53, phospho-ATM, and phospho-CHK1/2 (activated healthy T cells)	PPCA therapy eliminated only pathological CD4+ T cells	[52]
	CD4+ T	SLE	Increased γH2AX/defective repair after induced DNA damage		[54]
	CD4+ T	SLE	Elevated *GADD45A* mRNA expression and global DNA hypomethylation	Silencing of *GADD45A* increased DNA methylation and reduced T cell autoreactivity	[55]
	CD4+ T	RA	ATM and G2/M checkpoint insufficiencies due to ROS production deficiency	Pharmacologic interventions that restore ROS production (e.g., Menadione)	[59]
	CD4+ T resting	not assessed/p53 deficiencies	Unrepaired DNA upon induced DNA damage: a) deficient accumulation of 53BP and γH2AX foci and b) increased apoptosis via JNK/p73 pathway (non-DDR pathway)		[53]
	CD4+ T follicular	SLE	*Polb* decreased expression generated a lupus phenotype and increased GC CD4+ T follicular cells which were mainly apoptotic (CD4+).		[47]
	Tregs	MS, SLE, RA	Increased *H2AFx*, *TP53*, *CHK2*, *TP53BP1*/oxidative stress(mtROS)/cell death	Treg-specific scavenging of mtROS in vivo restrained DDR, reduced cell death and autoimmune responses	[60]
	B	SLE	*Polb* decreased expression generated a lupus phenotype, altered V(D)J, increased SHM and increased GC B cells.		[47]
	B naïve	RA with bone erosion	Low *ATM*, *MRE11A*, *NBS1*		[66]
	CD20+ B	RA or RA with bone erosion	Low phospho-ATM limited BCR, increased immunogenic B cells and secretion of RANKL and IL-6 cytokines.	Anti-CD20+ B cell–depleting biologic therapy (i.e., rituximab)	[66]
	B lymphoblastoid cell lines	SLE	Ineffective DNA repair mechanisms		[68]
	B subsets (rN, T3, aN, SM, DN2)	SLE	p53 pathway upregulated (all subsets); G2/M pathway upregulated (all subsets-DN2); G2/M pathway downregulated (DN2)		[69]
**Oher immune cells interacting with adaptive immune cells**	DCs	not assessed/high NLRP3 and Caspase 1	Reduced DDR and p53 induced apoptosis upon attenuation of Nlrp3 and Caspase 1	Decrease Nlrp3 and Caspase 1 expression	[71]
	DCs	not assessed/ATM expression defects	ATM inhibition delayed maturation, increased apoptosis and reduced T-cell activation.		[74]
	NK	not assessed/RAG deficiencies	RAG deficiency increased γH2AX and reduced *DNA-PKcs*, *Ku80*, *CHK2* and *ATM* expression levels.		[84]
	NK, γδ Τ, ΝΚΤ, T subsets	not assessed/aberrant expression of NKG2D receptor and ligands	Chronic activation of DDR as occurs in tumor cells may account for NKG2D ligand overexpression.		[91]

Tregs: Regulatory T cells; rN: resting naïve; T3: transitional 3, aN: activated naïve; SM: isotype-switched memory; DN2: double negative 2; DCs: dendritic cells; NK: natural killer cells; γδ Τ: gamma delta T cells; ΝΚΤ: natural killer T cells; HLH: hemophagocytic lymphohistiocytosis; SLE: systemic lupus erythematosus; MS: multiple sclerosis; RA: rheumatoid arthritis; DDR: DNA damage response; ROS: reactive oxygen species; mtROS: mitochondrial reactive oxygen species; PPCA: p53 potentiation with checkpoint abrogation; BCR: B-cell receptor; SHM: somatic hypermutation; GC: germinal center.

**Table 2:** Research Agenda.

1.	Assess alterations in the DDR and provide mechanistic insights for pathogenic cell populations during autoimmunity.
2.	Link the DDR molecules’ expression with specific cell behavior during autoimmune response.
3.	Investigate the effects of the DDR on immune cell interactions.
4.	Explore both the activated and total forms of the DDR proteins in autoimmunity.
5.	Distinguish between the beneficial and the detrimental outcomes of the DDR during immune responses.
6.	Examine the perturbation of the DDR components towards the suppression of distinct autoimmune responses.
7.	Generate animal models for autoimmune diseases that allow cell-targeted therapeutic approaches of the DDR.
8.	Extrapolate existing in vitro and in vivo research to autoimmune human diseases.
9.	Explore the potential therapeutic benefit of targeting the DDR pathway in cancers for autoimmunity and the role of the DDR-affecting cell-penetrating autoantibodies in autoimmunity to engineer therapeutic antibodies.

DDR: DNA damage response.

## Abbreviations

**Molecule**	**Detailed name or function**
AGO1	Protein argonaute-1
APEX1	Apurinic/apyrimidinic endodeoxyribonuclease 1 protein
ATM	Ataxia telangiectasia-mutated protein kinase
ATR	Ataxia telangiectasia- and Rad3-related protein kinase
AURKA	Aurora kinase A
BRCA1	Breast cancer type 1 susceptibility protein
BRCA2	Breast cancer type 2 susceptibility protein
CHK1	Checkpoint protein kinase 1
CHK2	Checkpoint protein kinase 2
DNA-PKcs	Catalytic subunit of DNA-dependent protein kinase
GADD45A	Growth arrest and DNA damage-inducible alpha gene
H2AFx	H2A histone family member X
HMGB1	High-mobility group protein B1
IFIT5	Interferon-induced protein with tetratricopeptide repeats 5
IFN-γ	Interferon gamma 1
IL-6	Interleukin 6
Ku	Dimeric protein complex: heterodimer of two polypeptides, Ku70 and Ku80
Ku70	Protein encoded by the XRCC6 gene
Ku80	Protein encoded by the XRCC5 gene
MAPKAPK3	MAP kinase-activated protein kinase 3
MDM2	E3 ubiquitin–protein ligase
MLH1	DNA mismatch repair protein
MRE11A	Meiotic recombination 11 homolog; double-strand break repair protein
MRN	Complex-interacting protein complex consisting of MRE11A, RAD51 and NBS1
NBS1	Nijmegen breakage syndrome protein 1; DNA repair and telomere maintenance protein
p53	Tumor protein encoded by TP53 gene
p53BP1	Tumor protein p53 binding protein 1 encoded by TP53BP1 gene
PADI4	Protein arginine deiminase type-4
PARP	Poly (ADP-ribose) polymerase family of proteins
PMS1	Protein homolog 1; mismatch repair system component
PMS2	Protein homolog 2; mismatch repair system component
POLB	DNA polymerase beta
RAD51	DNA repair protein homolog 1
RAG	Recombination-activating gene enzyme
RANKL	Receptor activator of nuclear factor kappa-Β ligand protein; member of the tumor necrosis factor (TNF) cytokine family
RGS3	Regulator of G-protein signaling 3
RPA	Replication protein A
TNF-α	Tumor necrosis factor alpha cytokine
TREX1	Three-prime repair exonuclease 1
UBE2S	Ubiquitin-conjugating enzyme E2 S
VRK1	Vaccinia related kinase 1
WEE1	G2 checkpoint kinase
WRN	Werner syndrome ATP-dependent helicase
γH2AX	Phosphorylated form of histone H2AX protein encoded by H2AFx gene
The UniProt Consortium: UniProt: the universal protein knowledgebase in 2021 [119]; GeneCards: the human gene database [120]	
